# The Prevalence of Programmed Death Ligand-1 (PD-L1) Expression in Non-Small Cell Lung Cancer: An Experience From Tertiary Care Hospital

**DOI:** 10.7759/cureus.72291

**Published:** 2024-10-24

**Authors:** Asmita Mehta, Lakshmi Priya V P, Lakshmi Teja, Krishna Pillai

**Affiliations:** 1 Respiratory Medicine, Amrita Institute of Medical Sciences, Amrita Vishwa Vidyapeetham, Kochi, IND

**Keywords:** anaplastic lymphoma kinase, epidermal growth factor receptor, lung cancer, non-small cell lung cancer, programmed cell death ligand 1

## Abstract

Background: The prevalence of programmed cell death ligand-1(PD-L1) expression in non-small cell lung cancer (NSCLC) within unselected populations remains a research topic, though PD-L1 expression is important in guiding treatment decisions.

Objectives: The study objective was to ascertain the prevalence of PD-L1 expression in patients of NSCLC and its association with clinicopathological characteristics and other gene mutations.

Methods: Samples from patients with NSCLCs were analyzed in the study to determine the expression of PD-L1 through immunohistochemistry (IHC) using rabbit anti-human PDL-1/CD274 monoclonal antibody. Correlation analysis was done using Pearson's correlation coefficient (r). The study analyzed the association between PD-L1 expression and clinicopathological features.

Results: A total of 245 consecutive patients (154 men with 62.9%) with NSCLCs were subjected to PD-L1 testing, and 30.6% (n=75) were identified to be positive. It was twice as prevalent in men than women, with 154 men and 91 females, respectively. The PD-L1 expression failed to demonstrate any statistical significance with age, gender, smoking status, type of materials, location of biopsy, Eastern Cooperative Oncology Group Performance Status, and Epidermal Growth Factor Receptor mutation. High PD-L1 expression with tumor proportion score (TPS ≥ 50) was observed in 38.8% of patients, and it was more prevalent in female patients 29 (38.7%), smokers 59 (78.7%), stage IV tumors 44 (58.7%), and stage III tumors 28 (37.3%).

Conclusions: Our study in Kerala showed PD-L1 expression in NSCLC patients, correlated with clinicopathologic factors. Males, COPD, ALK+, and advanced-stage tumors had higher expression. Females, smokers, poorly differentiated tumors, and stage IV tumors had high PD-L1.

## Introduction

The estimated number of new cancer cases in India for 2022 was 1,461,427, with a crude rate of 100.4 per 100,000 individuals. In India, one in nine people is expected to develop cancer in their lifetime. Lung cancer is the leading site of cancer in males, while breast cancer is the most common among females [1]. The economic review said the high prevalence of non-communicable disease control (NCD) in Kerala is attributed to drastic lifestyle changes, heavy dependency on alcohol and tobacco, an affinity for white-collar jobs, unhealthy eating patterns, low priority for physical exercise, and high levels of stress in all strata of the population. The study also showed that Kerala ranked second among overall cancer rates across India [2]. Late presentation of patients frequently leads to late-stage diagnoses of lung cancer, and Kerala is no different in this regard. Tobacco use, including smoking, is a major risk factor for most cancers, particularly lung cancer [2,3]. Non-small cell lung cancer (NSCLC) is one of the major causes of cancer-related fatalities globally. Due to limited treatment options, many patients are left with limited options, making it difficult to find effective methods of disease management [4].

Immunological checkpoints are immune-inhibitory pathways due to genetic changes in cancer cells. Programmed death-1 is a protein found on the surface of immune cells. It can interact with its ligand, programmed death ligand 1 (PD-L1). PD-L1 is expressed on the plasma membrane, the surface of secreted cellular exosomes, in cell nuclei, or as a circulating soluble protein. Interaction of PD-L1 with cells can lead to immune escape in cancer patients. Programmed cell death Ligand-1 is important in diagnosing tumor diseases, determining therapeutic effectiveness, and predicting patient outcomes. PD-L1 inhibitors have become an essential part of tumor immunotherapy. The detection of PD-L1 levels is crucial for precision cancer therapy [3]. PD-L1 is an example of an immune-inhibitory pathway that limits T-cell activation and proliferation when paired with tumor cells [5]. The introduction of immune checkpoint inhibitors, which focus on the programmed death-1 (PD-1) pathway, has transformed the treatment of non-small cell lung cancer (NSCLC). The expression of PD-L1 on tumor cells is considered an essential biomarker for the prediction of response to anti-PD-1/PD-L1 therapies in immunotherapy for NSCLC [6]. Certain anti-PD-1/PD-L1 medications, such as atezolizumab, pembrolizumab, and nivolumab, are authorized for the management of advanced non-small cell lung cancer [7]. A correlation between PD-L1 expression and a few genetic mutations e.g. epidermal growth factor receptor (EGFR), Anaplastic lymphoma kinase (ALK), and (Kristen Rat Sarcoma viral oncogene homolog) KRAS has been shown in a few studies [7].

The prevalence of PD-L1 expression in non-small cell lung cancer (NSCLC) within unselected populations remains a research topic, though PD-L1 expression is important in guiding treatment decisions. Understanding the range of PD-L1 expression levels in diverse NSCLC patients is necessary for improving patient outcomes and developing personalized treatment strategies. In recent years, innovations have been made in traditional immunoassay methods and the development of new immunoassays for PD-L1 detection [3]. The present study aimed to find out the expression of PD-L1 in patients with non-small cell lung cancer (NSCLC) from Kerala and its relationship to different clinical and pathological parameters, including gender, smoking status, metastasis, pleural effusion, and histopathological subtypes, along with gene mutations like epidermal growth factor receptor (EGFR) and anaplastic lymphoma kinase (ALK) [7].

## Materials and methods

Methods

We conducted an observational study at a tertiary care hospital in South India. The institute's Ethics Committee/Institutional Review Board (IRB) has approved this study (ECASM-AIMS-2022-109) based on the criteria of good clinical practice and the Declaration of Helsinki. Informed consent was obtained from all the patients before their inclusion in the study. All consecutive patients of ≥18 years with proven non-small cell lung carcinoma from November 2022 and November 2023 were studied.

A comprehensive clinical proforma was developed to gather patient clinical details and history, including age, gender, smoking status, cancer stage, biopsy location, and symptoms at the time of diagnosis. Biopsy and cytology samples were evaluated simultaneously for Epidermal Growth Factor Receptor (EGFR) using polymerase chain reaction (PCR) or gene sequencing for Anaplastic Lymphoma Kinase (ALK) through immunohistochemistry (IHC) or fluorescence in situ hybridization (FISH), and for PD-L1 expression via IHC. A standardized scoring system based on staining intensity and the percentage of tumor cells exhibiting membranous PD-L1 staining was employed to assess PD-L1 expression.

Study outcomes

The purpose of the study was to find out the prevalence of high PD-L1 expression. High PD-L1 expression is defined as tumors that express high amounts of PD-L1 (50% or greater) among non-small cell lung cancer (NSCLC) patients. We also observed the number of patients having a tumor percentage score (TPS) of 50% or higher on the PD-L1 test. The correlation between PD-L1 expression and clinicopathological parameters such as gender, smoking, cancer stage, location of biopsy, and histopathological examination, as well as epidermal growth factor receptor (EGFR) and anaplastic lymphoma kinase (ALK) mutations, were also analyzed.

Statistical analysis

A statistical analysis was conducted to determine the prevalence of programmed death ligand-1 (PD-L1) expression in the study population and to examine possible correlations between PD-L1 expression levels and clinical parameters. IBM Corp. Released 2011. IBM SPSS Statistics for Windows, Version 20.0. Armonk, NY: IBM Corp. was used to analyze the data after it was imported into Microsoft Excel 2010 (Microsoft Corporation, USA). Percentages as well as numbers were used to express the characteristics of the population. A descriptive analysis was performed on the incidence of PD-L1. The Pearson's correlation coefficient (r) approach was utilized to determine the pattern of correlation of PD-L1 and clinic-pathological characteristics. P-values <0.05 were considered as statistically significant. Multivariate analysis was done with the linear regression method, and variables of significance were found.

## Results

Among the unselected consecutive population of 245 non-small cell lung carcinoma (NSCLC) patients included in this study, programmed death ligand-1 expression was detected in 75 (30.6%) tumor samples. There were 154 (62.9%) men. The mean age was 65 ± 11.8 (SD). The most common co-morbidity was diabetes mellitus (DM) and hypertension, followed by coronary artery disease (CAD) and hypothyroidism (Table 1). The type of histology, site of biopsy, and histopathology specifications are also shown in Table 1. The distribution of PD-L1 expression levels was variable and ranged from 0% to 90%, with a 15% subset of patients exhibiting high PD-L1 expression (TPS >= 50%). Among them, 55 (73.3%) adenocarcinomas, 4 (5.3%) squamous cell carcinomas, and 100% each of large cell (n = 1) and spindle cell (n = 1) carcinomas expressed PD-L1. 

**Table 1:** The baseline characteristics of the study population. *ECOGPS: Eastern cooperative oncology group performance status, TPS: Tumor percentage score

Character	N Median (Percentage/Range)
Age	245, 65 ± 11.8 (25-88)
Gender: Male	154 (62.9)
Smoker	57 (23.4)
Comorbidities	N Median /Percentage
Coronary artery disease	34 (13.9)
Chronic liver disease	2 (0.8)
Chronic kidney disease	7 (2.9)
With diabetics	87 (35.5)
Hypertension	89 (35.5)
Others	202 (82.4)
Carcinoma type:	N Median/Percentage
Adeno carcinoma	193 (78.8)
Squamous cell carcinoma	22 (9.0)
Non-small cell lung cancer – not otherwise specified	12 (4.9)
Non-small cell lung cancer – others	18 (7.3)
Stage of carcinoma:	N Median/Percentage
I	3 (1.2)
II	10 (4.1)
III	63 (25.7)
IV	169 (69)
Previous Carcinoma	18 (7.3)
ECOG*	N Median/Percentage
0-3	192 (78.6)
4-5	52 (19.4)
Type of material	N Median/Percentage
Histology	168 (68.6)
Cytology with cell block	76 (31)
Others	1 (0.4)
Type of histology	N Median /Percentage
Bronchial/Transbronchial	36 (14.7)
Core needle	109 (44.5)
Metastasis form pleura/Pericardium	96 (39.2)
Metastasis from other than thorax	1 (0.4)
Location of biopsy	N Median /Percentage
Primary tumor in the lung	154 (63.1)
Metastasis from Lymph nodes N1-N3	56 (23)
Metastasis from Pleura/pericardium	11 (4.5)
Metastasis from outside the thorax	23 (9.4)
Programmed cell Death-Ligand 1: Positive	75 (30.6)
Anaplastic Lymphoma Kinase: Positive	25 (10.2)
PDL-1 TPS^*^ > 50%	37 (15)

Subgroup analyses based on clinical and pathological characteristics indicated that PD-L1 expression levels were significantly associated with the biopsy site. In the current study, none of the bronchial biopsy or transbronchial lung biopsy specimens showed PDL-1 positivity, while the positivity was high for core biopsy (lymph node or CT-guided biopsy from tumor) and pleural or pericardial metastasis (Table 2).

**Table 2:** The associations between PDL1 and other various clinical factors: multivariate analysis *ECOGPS: Eastern cooperative oncology group performance status, COPD: Chronic obstructive pulmonary disease, EGFR: Epidermal growth factor receptor, ALK: Anaplastic lymphoma kinase. The chi-square statistic is the difference in -2 log-likelihoods between the final model and a reduced model. The reduced model is formed by omitting an effect from the final model. The null hypothesis is that all parameters of that effect are 0. This reduced model is equivalent to the final model because omitting the effect does not increase the degrees of freedom.

variable	PD-L1				PD-L1 % (TPS >50%)			
	p-value	95.0% Confidence Interval	Standardized coefficients β	t	p-value	95.0% Confidence Interval	Standardized coefficient β	t
Gender	0.833	-0.107 - 0.133	0.13	0.211	0.661	-0.083-0.115	0.029	0.440
Smoking	0.043	-0.304 - 0.005	-0.142	-2.034	0.488	-0.164-0.080	-0.051	-0.695
Type of carcinoma	0.04	0.33 - 0.167	0.186	2.948	0.046	0.006-0.080	0.132	2.008
Type of material	0.141	-0.210- 0.030	-0.093	-1.476	0.562	-0.072-0.126	0.039	0.581
Location of biopsy	0.251	-0.096 - 0.025	-0.073	-1.152	0.056	-0.129-0.010	-0.126	-1.921
ECOGPS^*^	0.758	-0.046 - 0.033	-0.020	-0.309	0.851	-0.035-0.029	-0.013	-0.188
COPD^*^	0.001	0.178 - 0.519	0.284	4.022	0.011	0.033-0.311	0.189	2.580
Type of histology	0.010	0.025 - 0.187	0.166	2.586	0.209	0.026-0.108	0.086	1.261
EGFR^*^	0.122	-0.009 -0.078	0.096	1.552	0.762	-0.051-0.145	-0.020	-0.303
ALK^*^	0.015	0.046 - 0.418	0.153	2.458	0.044	0.000-0.299	0.131	2.021
Previous carcinoma	0.751	-0.255 -0.184	-0.020	-0.318	0.602	-0.132-0.226	0.035	0.522

The result of the multivariate analysis indicated that PD-L1 expression had a significant association with the type of histology, co-existing COPD, smoking status, and ALK positivity (Table 3). It is obvious that the same was true for high PD-L1 expression (TPS >=50%). Programmed death ligand-1 (PD-L1) positivity was seen in 30.6% (n=75) of patients. It was almost twice as prevalent in men when compared with women. High PD-L1 expression (TPS ≥ 50) was observed in 38.8% of patients, and it was more prevalent in 29 female patients (38.7%), smokers in 59 cases (78.7%), stage IV tumors in 44 cases (58.7%), and stage III tumors with 28 (37.3%) cases have high tumor expression. The PD-L1 expression failed to demonstrate any statistical significance with age, gender, smoking status, type of materials, location of biopsy, Eastern Cooperative Oncology Group Performance Status (ECOGPS), and epidermal growth factor receptor (EGFR) mutation.

**Table 3:** The associations between PDL1 and other various clinical factors. *PDL-1: Programmed cell death ligand-1, TPS: Tumor percentage score, ECOGPS: Eastern cooperative oncology group performance status, COPD: Chronic obstructive pulmonary disease, EGFR: Epidermal growth factor receptor, ALK: Anaplastic lymphoma kinase. Pearson Chi-Square was used for analysis. a. Not assuming the null hypothesis. b. Using the asymptotic standard error, assuming the null hypothesis. c. Based on normal approximation.

Variable	PDL-1*	PDL-1%
	p-value	(TPS* >50%)
		p-value
Gender	0.743	0.386
Smoking	0.635	0.508
Type of carcinoma	0.04	0.045
Stages of carcinoma	0.034	0.046
Type of materials	0.624	0.057
Location of biopsy	0.478	0.54
ECOGPS*	0.083	0.646
COPD*	0.002	0.067
Location of histology	0.904	0.434
Type of histology	<0.001	0.042
EGFR*	0.366	0.496
ALK*	0.016	0.062

## Discussion

Lung cancer is a diverse group of tumors that affects many people worldwide. Over 60% of cases are diagnosed after they are at a critical stage (III/IV), beyond the scope of surgical therapy. Chemotherapy and radiation are the main treatment options for such cases. Therapeutic interventions targeting driver mutations are now available for non-small cell lung cancer (NSCLC). Despite the advantages of targeted therapy, the overall 5-year survival rate for lung cancer remains below 20% [8]. To address this challenge, immune checkpoint inhibitors have emerged as a novel and promising strategy for treating non-small cell lung cancer (NSCLC).

Programmed cell death ligand-1 (PD-L1) is a transmembrane protein expressed at low levels by a variety of healthy cell types, including immune, vascular, endothelial, and epithelial cells throughout different organ systems [9]. Normal T-lymphocytes also express PD-L1 on their surface; however, elevated levels of expression are observed in certain neoplasms [10]. Tumor cells are shielded from immune attack when programmed cell death protein-1 (PD-1) binds to programmed cell death ligand-1 (PD-L1). This results in the formation of a protective barrier on the tumor cell surface [9]. Immune modulation through checkpoint inhibition has shown promising results in various studies of human cancers, with several drugs targeting PD-L1 and PD-1 either approved or in late-stage development [9-13]. We assessed the frequency and pattern of PD-L1 expression in a cohort of NSCLC, comparing it with known oncogenic drivers and clinicopathological factors. The median age of the study cohort was 65 years, with age ranging from 25 to 88 years. This result aligns with previous research, as indicated in Table 4 [14-17]. 

**Table 4:** Comparison of factors associated with PD-L1 expression in the current study with previously published studies. *PD L-1: Programmed cell death-ligand 1

Study/author	n	PD L-1* positivity	Factors associated with programmed death-ligand 1 expression
Guldhammer et al. [14]	791	30% (240)	Diagnosis (adenocarcinoma) and stage (higher stage) are statistically significant factors
Kumar et al. [15]	101	33.66% (34)	Men, smokers, and patients with advanced cancer stages III or IV have all been linked to PD L1 positive
Ekta et al. [16]	252	53.2% (134)	Higher rates of high PD-L1 expression were seen in female patients, those over 60, smokers, and individuals with poorly differentiated stage IV tumors (28.2%)
Bahnassy et al. [17]	70	87.14% (61)	Male, advanced-stage tumor and Lymph node metastasis showed association
Mehta et al. (present study)	245	30.6% (75)	PDL1 positivity was reported in stage III and stage IV, adenocarcinoma, chronic obstructive pulmonary disease, anaplastic lymphoma kinase positivity

In our study, a male preponderance of 62.9% (n = 154) was observed with a male-to-female ratio of 2:1, and it resonates with the findings of other published studies (14-17). The PD-L1 immunopositivity rate in our cohort of NSCLC patients was 30.6% (n = 75) as seen in similar other studies where the positivity ranged from 30% to 87% [15-19]. A few Indian studies on the PD-L1 research in NSCLC by Kumar et al., Domadia et al., and Vallonthaiel et al. have reported positivity rates of 33.66% (34/101), 47% (63/134), and 27% (24/89) [15,19-22]. However, as in other studies, neither age nor gender showed any significant difference among the PD-L1-positive or PD-L1-negative expressions in our study [18,19]. Our cohort consisted of 193 (78.8%) adenocarcinomas and 22 (9%) squamous cell carcinomas. Among them, 55 (73.3%) adenocarcinomas, 4 (5.3%) squamous cell carcinomas, and 100% each of large cell (n = 1) and spindle cell (n = 1) carcinomas expressed PD-L1. This is similar to other studies where PD-L1 expression was higher in squamous cell carcinoma (44%-50% with squamous cell carcinoma and 41%-50.5% with adenocarcinoma) in comparison with other types of NSCLC (18-22). High PD-L1 expression (TPS ≥ 50) was observed in 75 (30.6%) patients.

Pawelczyk et al. reported results with ≥50% PD-L1 expression in 7.4% and 10.6% patients versus 32.4% in a study by Kumar et al., as shown in Table 4 [14-17,22]. The high-positive tumors included 73.3% (55/193) adenocarcinoma and 5.3% (4/22) squamous cell carcinoma. It was more prevalent in 29 female patients in 29 cases (38.7%), smokers in 59 cases (78.7%), and stage IV tumors in 44 cases (58.7%). When the TPS ≥ 50% PD-L1 score was taken into account, stage III, with 28 (37.3%) cases, and stage IV, with 44 (58.7%) cases, had more PD-L1 expression than the other stages. These findings were comparable to other studies where PD-L1 positivity was commoner with stage IV with 87 (86.14%) cases and poorly differentiated tumors with 3 (35.6%) cases [15].

Extensive research has provided evidence linking mutations in the Anaplastic Lymphoma Kinase (ALK) and the epidermal growth factor receptor (EGFR) to the upregulation of PD-L1 expression, which is mediated by many oncogenic signaling pathways [7,23-26]. Researchers have shown up to 72% of EGFR-mutated and 78% of ALK-rearranged tumors in PD-L1-positive patients [15].

The findings of this investigation contribute to the increasing body of knowledge on the frequency of PD-L1 expression in non-small cell lung cancer (NSCLC) and the implications for patient care. The discovery of various PD-L1 expression levels highlights the significance of thorough biomarker testing in correctly classifying patients for immunotherapy. One indication of the possible advantages of immune checkpoint inhibitor therapy for a subset of NSCLC patients is the presence of elevated PD-L1 expression in these patients. According to the present investigation, there is a statistical correlation between PD-L1 expression and factors such as tumor stage, histological type, co-occurrence of COPD, and ALK-positive. The data presented in Table 4 compares the variables linked to PDL1 positive in the current investigation with previously published research.

Detecting PD-L1 expression in cancer cells is essential for guiding immunotherapy and optimizing cancer treatment strategies. By measuring PD-L1 levels in tumors, healthcare providers can better predict how patients will respond to immunotherapy, allowing for more personalized treatment plans that cater to individual needs. This approach to precision medicine can lead to better treatment outcomes, fewer side effects, and possibly lower healthcare costs. Additionally, understanding PD-L1 expression can guide doctors in making better clinical decisions, refine treatment strategies, and support research focused on developing more effective therapies for cancer patients. Overall, detecting PD-L1 is essential for advancing public health efforts and improving outcomes in cancer care [3].

Our study is the first study illustrating the frequency of PD-L1 expression in newly diagnosed NSCLC cases in Kerala. The fact that PD-L1 evidence was examined in conjunction with ALK and EGFR in every newly diagnosed NSCLC patient underscores the significance of our study. Our findings will assist in identifying regional variations in PD-L1 expression in the Indian population, as the previously published study only depicts the northern portion of the country. The cohort's size and selection are limited because they came from a tertiary healthcare center and could not be entirely representative of the broader population. Analyzing a large clinical patient sample could yield different results; however, it is important to consider the impact of selection bias.

## Conclusions

To the best of our knowledge, this is the first study on the prevalence of programmed death ligand-1 (PD-L1) expression among non-small cell lung cancer patients of Kerala. It is also the largest single-center study demonstrating PD-L1 expression in NSCLC patients and its comparison with clinic-pathologic parameters. PD-L1 expression was significantly associated with male gender, co-existing chronic obstructive pulmonary disease (COPD), anaplastic lymphoma kinase (ALK) positivity, and advanced-stage tumors. High PD-L1 expression was more prevalent in smokers and advanced stage IV tumors (58.7%). As PDL1 is a potential predictive marker stratifying patients who benefit from PD-L1 pathway-targeted therapy, understanding the relationships among factors affecting PD-L1 may aid in optimizing therapy in lung cancer patients.
